# Proton pump inhibitor use and risk of hip fracture in patients with type 2 diabetes

**DOI:** 10.1038/s41598-020-70712-9

**Published:** 2020-08-21

**Authors:** Ya-Shuan Chou, He-Jiun Jiang, Chung-Hwan Chen, Pei-Shan Ho, Tien-Ching Lee

**Affiliations:** 1 Orthopaedic Research Center, College of Medicine, Kaohsiung Medical University Hospital, Kaohsiung Medical University, No.100, Tzyou 1st Road, Kaohsiung, 807 Taiwan; 2 Regenerative Medicine and Cell Therapy Research Center, Kaohsiung Medical University, Kaohsiung, Taiwan; 3 Division of Endocrinology and Metabolism, Department of Internal Medicine, E-Da Hospital, Kaohsiung, Taiwan; 4 Department of Orthopedics, Kaohsiung Municipal Ta-Tung Hospital, Kaohsiung Medical University Hospital, Kaohsiung Medical University, Kaohsiung, Taiwan; 5 Department of Orthopaedics, College of Medicine, Kaohsiung Medical University Hospital, Kaohsiung Medical University, Kaohsiung, Taiwan; 6 Faculty of Dental Hygiene, College of Dental Medicine, Kaohsiung Medical University, Kaohsiung, Taiwan; 7 Graduate Institute of Medicine, College of Medicine, Kaohsiung Medical University Hospital, Kaohsiung Medical University, Kaohsiung, Taiwan

**Keywords:** Type 2 diabetes, Osteoporosis, Risk factors, Outcomes research

## Abstract

Type 2 diabetes mellitus (T2DM) is associated with a high rate of comorbidity, including osteoporosis and peptic ulcers. Proton pump inhibitors (PPIs) are a group of acid-suppressing drugs commonly used for treating peptic ulcers. However, observational studies have reported an association between PPI therapy and osteoporotic fractures. This study investigated the association between PPI use and hip fracture (HFx) among patients with T2DM. We conducted this population-based propensity-matched retrospective cohort study using the National Health Insurance Research Database in Taiwan. Patients newly diagnosed with T2DM between 2000 and 2008 were identified. After excluding those who previously used PPIs or suffered HFx, 398,885 patients were recruited (44,341 PPI users; 354,544 non-users). HFx risk data from 2000 to 2013 were collected to calculate the cumulative rate of HFx in these two groups. Sensitivity analyses were conducted to evaluate the effects of PPI dose. After propensity score matching of 1:4, 44,431 and 177,364 patients were assigned to the PPI user and non-user groups, respectively. PPI user group showed an increased risk of HFx with an adjusted hazard ratio of 1.41 (95% CI 1.29–1.54) without dose–response relationship. Thus, there is an increased risk of HFx in patients with T2DM receiving long-term PPI treatment.

## Introduction

Type 2 diabetes mellitus (T2DM) is a common metabolic disease worldwide. In Taiwan, diabetes is one of the 10 leading causes of death. The global prevalence of diabetes mellitus has more than doubled in the past 30 years^1^. Common comorbidities of T2DM include osteoporosis^2–5^, peptic ulcer^6–8^, heart and blood vessel disease, diabetic neuropathy, diabetic retinopathy, liver cirrhosis, and delayed wound healing^9^. The risk of osteoporosis and associated fragility fracture is increased 1.2-fold in patients with T2DM^5^. Among associated fragility fractures, hip fracture (HFx) is one of the most serious, with a 1-year mortality rate ranging from 2.4% in Japan to 34.8% in Hungary^10^.

Previous studies have shown a higher incidence of perforated peptic ulcer disease and related mortality rates in patients with T2DM compared with those in patients without the disease^6–8^. Proton pump inhibitors (PPIs) are a group of acid-suppressing drugs commonly used in the treatment of peptic ulcers. However, several studies have reported an association between treatment with PPIs and risk of fractures, especially HFx^11–15^. Although most of these studies report a higher risk of fractures in patients with high-dose and long-term use of PPIs^11,12,16,17^, the association and mechanisms involved remain controversial. The literature lacks information about risk of HFx in patients with T2DM receiving PPI treatment.

The present study was a population-based cohort study using data from the National Health Insurance Research Database (NHIRD) at the National Health Research Institutes (NHRI) in Taiwan to elucidate the association between use of PPIs and risk of HFx among patients with T2DM.

## Materials and methods

### Data sources

In 1995, Taiwan launched a single-payer mandatory enrollment National Health Insurance (NHI) program. Taiwan’s established NHRI has continued to maintain the NHIRD in Taiwan since 2002. At the end of 2018, the coverage rate was more than 99.9% of Taiwan’s population^18^.

The NHIRD includes all claims data of the NHI program and makes these data available to scientists in Taiwan for research purposes. The NHIRD is an individual-level claim database comprising data on sex, date of birth, diagnostic codes, medical records of clinical visits, hospitalizations, diagnosis codes, prescriptions, procedures/surgeries, and expenditures.

We conducted a population-based propensity-matched retrospective cohort study using data from the Longitudinal Cohort of Diabetes Patients (LHDB 2000), which contains random samples from 120,000 patients each calendar year with newly diagnosed diabetes mellitus enrolled from the 2000 Registry of NHI Beneficiaries (a total of 1.68 million enrollees from 2000 to 2013).

### Identification of cases

**Figure 1:**
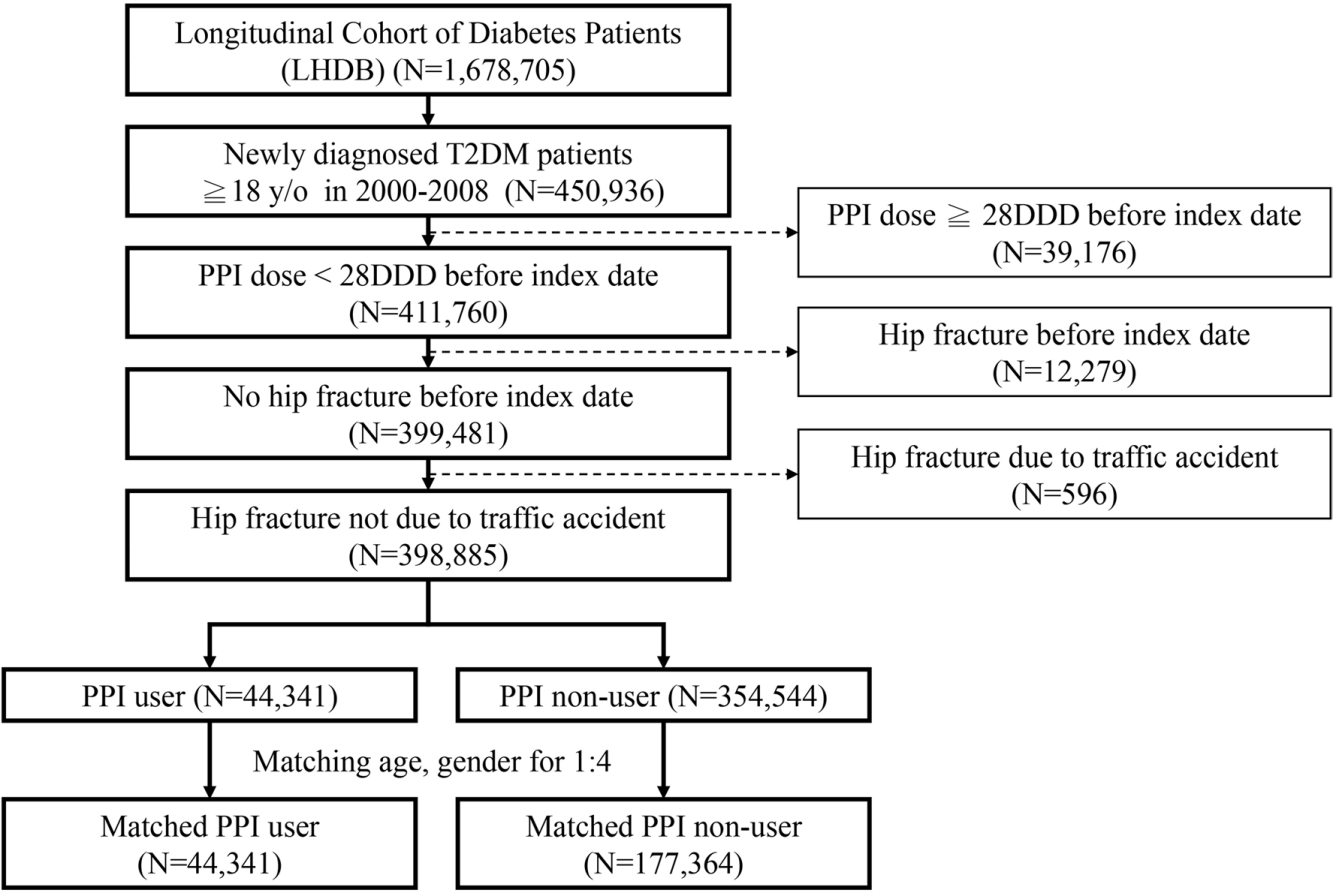
Flow diagram of the present study from the National Health Insurance Research Database in Taiwan.

Patients aged ≥ 18 years with newly diagnosed T2DM between 2000 and 2008 were identified. Those who previously used PPIs [defined daily dose (DDD) ≥ 28] or suffered HFx prior to the index date were excluded. In total, 399,481 patients were eligible for analysis (Fig. 1).

During the longitudinal study period, we excluded fractures associated with HFx due to traffic accident. A final total of 398,885 patients were recruited, including 44,341 PPI users and 354,544 non-users.

### Propensity score matching

Propensity score matching was performed to minimize selection bias by balancing baseline characteristics, including age and gender. The propensity score was calculated for each PPI user and non-user using all covariates by logistic regression model. The optimal ratio from the analysis of variable multiple pairing was 1:4.

### Outcome and comorbidities

Primary outcome was defined as any new diagnosis of HFx (ICD-9-CM codes 820–820.9) with medical codes for internal fixation or hemiarthroplasty (ICD-9-CM codes 79.15, 79.35, and 81.52). Data on HFx during the period from 2000 to 2013 were collected to calculate the cumulative rate of HFx in the PPI and non-PPI groups. Patients in both the PPI and non-PPI groups were followed up until either the endpoint of study (December 31, 2013) or the occurrence of one of the following events: primary outcome, censoring due to loss to follow-up, or withdrawal from insurance. Hazard ratios (HRs) were calculated for risk of HFx according to PPI use or non-use. Sensitivity analyses were conducted to evaluate the dose effects of PPI treatment.

Charlson comorbidity index (CCI)^19^ scores were used to assess the severity of comorbidities, including hypertension, stroke, asthma, chronic obstructive pulmonary disease (COPD), myocardial infarction, chronic heart failure, dementia, depression, schizophrenia, chronic renal failure, peripheral vascular disease, and rheumatoid arthritis. CCI scores were then categorized as 0, 1, 2, or ≥ 3.

Previous use of medications such as nonsteroidal anti-inflammatory drugs (NSAIDs), corticosteroids, anticoagulants, diuretics, antipsychotic, thyroxine, hormone therapy, statins, antihypertensive, sedatives, and bisphosphonates by patients for at least 3 years was defined as long-term use.

### Statistical analysis

Data on HFx during the period from 2000 to 2013 were collected to evaluate the cumulative rate of HFx in the PPI and non-PPI groups. Student’s *t* test was used to analyze the continuous variables and Chi-squared test was used for categorical variables. The cumulative incidences of HFx according to PPI use and cumulative dose were estimated using the Kaplan–Meier method, and differences between the cumulative incidence curves were compared using log-rank test. Cox proportional hazards models were used to calculate the HRs and 95% confidence intervals (CIs) of HFx in the PPI user and non-user groups in patients with T2DM over a 5-year period. HR was calculated for risk of HFx according to PPI use or non-use. Sensitivity analyses were conducted to evaluate the dose effects of PPIs. This study complied with the Helsinki Declaration. The data in this study were collected with the approval of the Institutional Review Board of Kaohsiung Medical University Hospital (KMUHIRB-EXEMPT(II)-20170017) after obtaining informed consent.

## Results

### Patient characteristics

After propensity score matching of 1:4, 221,795 patients with T2DM were enrolled. Among these, 44,431 and 177,364 patients were assigned to the PPI user and non-user groups, respectively. The matching procedure achieved a good balance of baseline characteristics between the two groups, without significant between-group differences related to distribution of gender and age category (Table 1). Furthermore, the prevalence of some comorbidities, including hypertension, stroke, asthma, COPD, myocardial infarction, chronic heart failure, depression, chronic renal failure, and rheumatoid arthritis, were higher in the PPI user group compared with those in the non-user group. Use of prescribed medications, including NSAIDs, corticosteroids, anticoagulants, diuretics, antipsychotic, thyroxine, statins, antihypertensive, and sedatives, was also significantly greater in the PPI user group compared with that in the non-user group (Supplementary Table 1).

**Table 1:** Demographics of patients with and without PPI use after propensity score matching.

	PPI user	PPI non-user	*P* value
	Mean ± SD/(N,%)	Mean ± SD/(N,%)	
Case no	44,341	177,364	
**Gender**			
Female (N,%)	18,979 (42.80%)	75,916 (42.80%)	1.000
Male (N,%)	25,362 (57.20%)	101,448 (57.20%)	
Age (Mean ± SD)	59.23 (± 12.56)	58.95 (± 12.71)	< 0.001
**Age category (N,%)**			
50–59	5679 (12.81%)	22,716 (12.81%)	1.000
60–69	11,672 (26.32%)	46,688 (26.32%)	
70–79	11,853 (26.73%)	47,412 (26.73%)	
> 80	15,137 (34.14%)	60,548 (34.14%)	
Insurance premium (NTD/month)	16,536 (± 19,082)	16,313 (± 18,831)	0.028
**Insurance premium category**			
Dependent	11,942 (26.93%)	49,355 (27.83%)	0.002
< 20,000	17,492 (39.45%)	68,832 (38.81%)	
20,000–39,999	10,148 (22.89%)	40,173 (22.65%)	
> 40,000	4759 (10.73%)	19,004 (10.71%)	
CCI score	1.67 (± 1.62)	1.32 (± 1.44)	< 0.001
**CCI score category**			
0	11,012 (24.83%)	56,851 (32.05%)	< 0.001
1	13,325 (30.05%)	60,563 (34.15%)	
2	9684 (21.84%)	32,160 (18.13%)	
3+	10,320 (23.27%)	27,790 (15.67%)	

*SD* standard deviation, *NTD* New Taiwan dollar, *CCI* Charlson comorbidity index.

### Cox proportional hazards regression model

Cox proportional hazards regression analysis highlighted several risk factors for HFx in patients with T2DM (Table 2). All the covariables, including comorbidities and medication listed in Supplementary Table 1 were adjusted for. Use of PPIs was associated with an increased risk of HFx, with an adjusted HR of 1.41 (95% CI 1.29–1.54; *P* < 0.001). During the follow-up period, 683 (1.54%) patients in the PPI group suffered HFx, compared with 1808 (1.02%) in the PPI non-user group. Multiple regression models showed a lower risk of fracture in males compared with that in females, with an adjusted HR of 0.65 (95% CI 0.60–0.70; *P* < 0.001). In addition to female gender, old age (≥ 70 years), low insurance premium [< 20,000 NTD (New Taiwan Dollar)/month], and comorbidity (CCI ≥ 3) were statistically significant independent risk factors for HFx (*P* < 0.001).

**Table 2:** Cox proportional hazards regression model of hip fracture.

	Fracture no	Crude HR (95% CI)	*P* value	Adjust HR (95% CI)	*P* value
PPI non-user(Ref)	1808 (1.02%)	1 (Ref.)		1 (Ref.)	
PPI user	683 (1.54%)	1.52 (1.39–1.66)	< 0.001	1.41 (1.29–1.54)	< 0.001
**Baseline patient demographic characteristics**					
Gender					
Female (Ref.)	1,443 (1.52%)	1 (Ref.)		1 (Ref.)	
Male	1,048 (0.83%)	0.55 (0.51–0.60)	< 0.001	0.65 (0.60–0.70)	< 0.001
Age categories					
50–59 (Ref.)	39 (0.14%)	1 (Ref.)		1 (Ref.)	
60–69	106 (0.18%)	1.33 (0.92–1.92)	0.130	1.31 (0.90–1.88)	0.156
70–79	329 (0.56%)	4.12 (2.96–5.74)	< 0.001	3.60 (2.58–5.02)	< 0.001
> 80	2,017 (2.66%)	21.59 (15.72–29.63)	< 0.001	15.61 (11.31–21.52)	< 0.001
**Insurance premium (NTD/month)**					
< 20,000 (Ref.)	1,187 (1.38%)	1 (Ref.)		1 (Ref.)	
Dependent	967 (1.58%)	1.14 (1.05–1.24)	0.002	0.88 (0.81–0.96)	0.005
20,000–39,999	306 (0.61%)	0.42 (0.37–0.48)	< 0.001	0.72 (0.63–0.82)	< 0.001
> 40,000	31 (0.13%)	0.09 (0.06–0.13)	< 0.001	0.33 (0.23–0.47)	< 0.001
**CCI score**					
0 (Ref.)	530 (0.78%)	1 (Ref.)		1 (Ref.)	
1	662 (0.90%)	1.15 (1.03–1.29)	0.016	0.98 (0.87–1.10)	0.730
2	543 (1.30%)	1.71 (1.52–1.92)	< 0.001	1.11 (0.98–1.26)	0.101
3+	756 (1.98%)	2.86 (2.56–3.19)	< 0.001	1.30 (1.14–1.49)	< 0.001

*95% CI* 95% confidence interval, *HR* relative hazard ratio, *Ref.* reference.

The results of the comorbidity-stratified and medication-use analysis of HFx risk showed that stroke and chronic renal failure patients with T2DM showed an increased risk of HFx, with adjusted HRs of 1.84 (95% CI 1.65–2.05, *P* < 0.001) and 2.01 (95% CI 1.58–2.57; *P* < 0.001), respectively. Hypertension, asthma, COPD, myocardial infarction, chronic heart failure, depression, and rheumatoid arthritis were not associated with increased risk of HFx after adjustment for other characteristics. Although a higher proportion of PPI users used other medications (Table 2), only use of anticoagulants and sedatives were associated with an increased risk of HFx after adjustment for characteristics (Supplementary Table 2). The adjusted HRs of HFx in patients with T2DM using anticoagulants and sedatives were 1.26 (95% CI 1.13–1.40; *P* < 0.001) and 1.33 (95% CI 1.19–1.48, *P* < 0.001), respectively. Patients who used statins had a decreased risk of HFx, with an adjusted HR of 0.61 (95% CI 0.52–0.70, *P* < 0.001).

### Association between risk of hip fracture and PPI use

**Figure 2:**
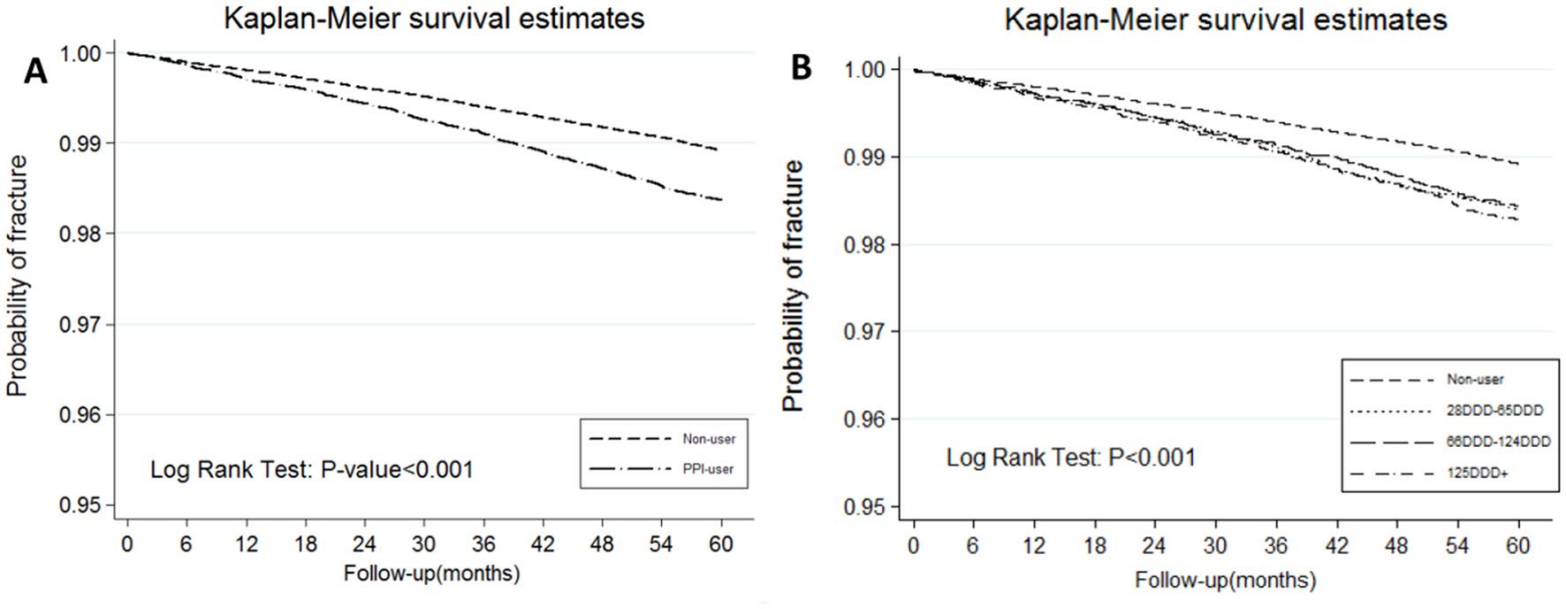
Kaplan–Meier curves showing the cumulative incidence of HFx for 5 years following diagnosis of type 2 diabetes according to PPI use (**A**) and cumulative dose (**B**).

Kaplan–Meier curves revealed the time-to-readmission events for cumulative incidence of fracture between the PPI user and non-user groups after a 5-year follow-up. The PPI group showed a significantly higher incidence of HFx than did the non-PPI group (incident rate, 3.27 vs. 2.16, respectively; log-rank test, *P* < 0.001; Fig. 2A).

We also evaluated the relationship between PPI dose and HFx (Table 3). The World Health Organization defines DDD as the assumed average maintenance dose per day for a drug used for its main indication in adults. The cumulative DDDs were estimated as the sum of the dispensed DDD of any PPI during 60 months. The PPI group was categorized into four subgroups: non-use (< 28 DDD), 28–65 DDD, 66–124 DDD, and > 125 DDD. The adjusted HRs of the 28–65 DDD, 66–124 DDD, and > 125 DDD subgroups were 1.46 (95% CI 1.27–1.68; *P* < 0.001), 1.39 (95% CI 1.21–1.60; *P* < 0.001), and 1.39 (95% CI 1.21–1.59, *P* < 0.001), respectively. Analysis of each event showed no significant differences in the dose–response relationship between PPI use and risk of HFx (log-rank test, *P* > 0.05; Fig. 2B).

**Table 3:** Risk of hip fracture according to cumulative dose of PPI.

PPI use	N	Fracture no	Crude HR (95% CI)	*P* value	Adjust HR (95% CI)	*P* value
Non-user (< 28 DDD)	177,364	1808 (1.02%)	1 (Ref.)		1 (Ref.)	
28–65 DDD	14,985	222 (1.48%)	1.49 (1.29–1.71)	< 0.001	1.46 (1.27–1.68)	< 0.001
66–124 DDD	14,608	218 (1.49%)	1.46 (1.27–1.68)	< 0.001	1.39 (1.21–1.60)	< 0.001
> 125DDD	14,748	243 (1.65%)	1.60 (1.40–1.83)	< 0.001	1.39 (1.21–1.59)	< 0.001

*95% CI* 95% confidence interval, *HR* relative hazard ratio, *Ref.* reference, *DDD* defined daily dose, *PPI* proton pump inhibitors.

## Discussion

This is the first population-based cohort study to evaluate the impact of PPI use on risk of HFx in patients newly diagnosed with T2DM. Predictors of HFx included well-known risk factors such as older age, female gender, and higher CCI score, which may reflect a more fragile medical state and higher risk of osteoporosis, as previously reported. We adjusted for multiple confounding factors to evaluate the effect of PPIs on risk of HFx in patients with T2DM. In patients with T2DM, PPI use was a significant and independent predictor of HFx, with an adjusted HR of 1.41. However, we did not observe a dose–response relationship between risk of HFx and dose of PPI used. This is in contrast to other studies^11–15^, which demonstrated an association between dosage of PPI and HFx was dose-dependent. This difference might be explained by the use of different statistical analysis methods or the susceptibility of T2DM patients to HFx^5^. Further research is needed to clarify the safety margin of PPI to avoid the risk of HFx.

Previous studies reported an elevated risk of subsequent fracture development after PPI therapy. Several possible relationships between fracture and PPI use have been reported, including postmenopausal women^20,21^, long-term use of PPIs^11^, history of smoking^14^, and conditions such as stroke^22^, Alzheimer’s disease^23^, and hemodialysis^24^. The present study revealed that stroke and chronic renal failure were associated with higher risk of HFx in patients with T2DM after adjustment. Our findings are supported by the findings of previous studies. Stroke is associated with an increased risk of falls and fractures^25^, and patients with chronic renal failure have an increased risk of fractures due to disordered mineral and bone metabolism^26^.

**Figure 3:**
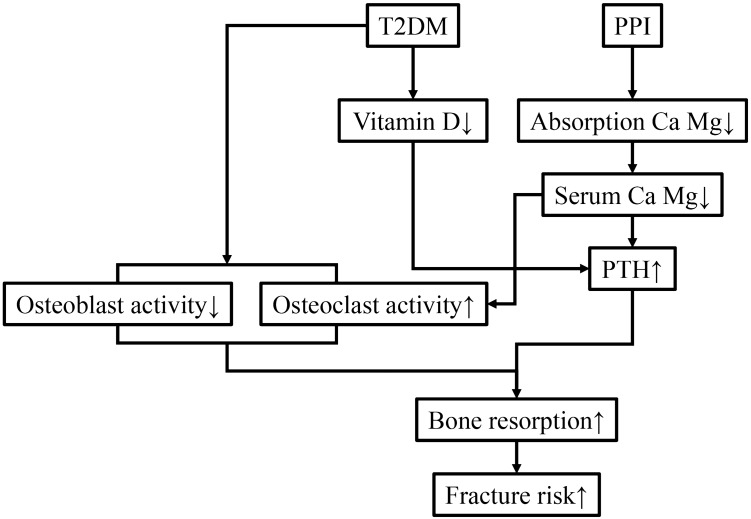
Flow chart showing the systemic effects of type 2 diabetes and PPIs that elevate the risk of fracture. ↑, increase; ↓, decrease; Ca, calcium; Mg, magnesium; PTH, parathyroid hormone.

Patients with T2DM have an increased risk of fracture due to falls related to nervous and vascular disease^25,27^ as well as changes in the microarchitecture that decrease bone strength and quality^28,29^. The effects of bone structure due to use of PPIs may be linked to malabsorption of calcium, hypergastrinemia, hypochlorhydria, hyperparathyroidism, and regulation of bone cells^15,30^. Previous studies have suggested that use of PPIs leads to increased gastric pH, and prolonged hypochlorhydria may reduce calcium ionization and affect intestinal absorption^15,31,32^. Low circulating calcium triggers secretion of parathyroid hormone (PTH) from the parathyroid gland to enhance bone resorption. PPI users had significantly higher PTH levels compared with that of non-users^33,34^. However, T2DM is also associated with impaired calcium metabolism, which may increase bone fragility^35^. Since PTH elevation is associated with abnormalities in glucose metabolism^4,36^, the etiology and link between PPI-induced HFx and T2DM should be examined. On the other hand, PPIs influence osteoblast and osteoclast activity, and regulate bone resorption^37^. Treatment using PPIs decreased gene expression of type I collagen, alkaline phosphatase, and bone morphogenetic protein 2 in Hematopoietic stem cells^38^. Moreover, T2DM is associated with impaired osteoblast differentiation and activity^39,40^. Since PPI users and patients with T2DM show increased risk of HFx, use of PPIs may lead to deterioration of the bone microstructure and strength, and increase the risk of fracture in patients with T2DM. Figure 3 summarizes the process of PPI-induced bone fracture and its association with T2DM.

Although our analysis was adjusted for comorbidity-stratified analysis and medication use, it is possible that these adjustments did not incorporate all the health conditions influencing PPI use. Consistent with previous studies, stroke was a risk factor for falls^41^ and was associated with increased risk of HFx. Elderly patients with diabetes were more likely to use sedatives, which was also associated with an increased risk of fall^3^. Furthermore, long-term users of anticoagulants often have a history of cerebrovascular accident, which may increase their likelihood of HFx due to an unsteady gait (Supplementary Table 2).

The present study has several limitations. First, the NHI database was not designed for academic research; hence, miscoding of diagnoses may have occurred. However, the coding error could be compensated for using medication codes for DM control and procedure codes for HFx (internal fixation or hemiarthroplasty). Second, the NHI database does not include patients’ functional status, compliance, personal habits (e.g., smoking, alcohol use), severity of comorbidities, nutritional status, biochemical data (e.g., hemoglobin A_1C_), time to surgery, and quality of postoperative care. Thus, it was not possible to show a dose response related to residual confounding or effect modification. Third, although we were unable to rule out the possibility that the dose–response relationship analyses may have been confounded by immortal time bias, our study revealed that use of PPIs was associated with an increased risk of HFx.

In conclusion, patients with T2DM using PPIs have an increased risk of HFx. Although unknown confounding factors may exist, these findings may provide a valuable basis for future prospective studies to investigate the relationship between PPI use and risk of HFx.

## Supplementary information

Supplementary file1

Supplementary file2
