# Supplementary material for: Proton pump inhibitor use and risk of hip fracture in patients with type 2 diabetes
Source: Sci Rep. 2020 Aug 21;10:14081. doi: 10.1038/s41598-020-70712-9 (PMC7443131; doi:10.1038/s41598-020-70712-9)
Supplement: Supplementary file 1 — Supplementary file1 [file 41598_2020_70712_MOESM1_ESM.pdf]

Supplementary Table 1. Comorbidities and medication use in patients with and without PPI use

	Before matching			After matching		
	PPI user	PPI non-user	P-value	PPI user	PPI non-user	P-value
	Mean ± SD/(N,%)	Mean ± SD/(N,%)		Mean ± SD/(N,%)	Mean ± SD/(N,%)	
**Comorbidity**						
Hypertension	19,979 (45.06%)	137,597 (38.81%)	<0.001	19,979 (45.06%)	74,958 (42.26%)	<0.001
Stroke	3,462 (7.81%)	20,459 (5.77%)	<0.001	3,462 (7.81%)	12,015 (6.77%)	<0.001
Asthma	1,872 (4.22%)	10,170 (2.87%)	<0.001	1,872 (4.22%)	5,645 (3.18%)	<0.001
COPD	4,762 (10.74%)	26,343 (7.43%)	<0.001	4,762 (10.74%)	14,813 (8.35%)	<0.001
Myocardial Infarction	847 (1.91%)	4,761 (1.34%)	<0.001	847 (1.91%)	2,755 (1.55%)	<0.001
Chronic Heart Failure	1,057 (2.38%)	5,366 (1.51%)	<0.001	1,057 (2.38%)	3,169 (1.79%)	<0.001
Dementia	36 (0.08%)	159 (0.04%)	0.001	36 (0.08%)	102 (0.06%)	0.074
Depression	291 (0.66%)	1,331 (0.38%)	<0.001	291 (0.66%)	701 (0.40%)	<0.001
Schizophrenia	257 (0.58%)	3,341 (0.94%)	<0.001	257 (0.58%)	1,460 (0.82%)	<0.001
Chronic renal failure	683 (1.54%)	2,489 (0.70%)	<0.001	683 (1.54%)	1,391 (0.78%)	<0.001
PVD	317 (0.71%)	1,860 (0.52%)	<0.001	317 (0.71%)	1,034 (0.58%)	0.001
Rheumatoid arthritis	299 (0.67%)	1,550 (0.44%)	<0.001	299 (0.67%)	787 (0.44%)	<0.001
**Medication**						
NSAID	24,774 (55.87%)	174,387 (49.19%)	<0.001	24,774 (55.87%)	87,067 (49.09%)	<0.001
Corticosteroids	14,647 (33.03%)	100,392 (28.32%)	<0.001	14,647 (33.03%)	50,660 (28.56%)	<0.001
Anticoagulants	4,805 (10.84%)	26,932 (7.60%)	<0.001	4,805 (10.84%)	14,946 (8.43%)	<0.001
Diuretics	15,799 (35.63%)	102,536 (28.92%)	<0.001	15,799 (35.63%)	54,757 (30.87%)	<0.001
Antipsychotic	4,063 (9.16%)	23,351 (6.59%)	<0.001	4,063 (9.16%)	11,575 (6.53%)	<0.001
Thyroxine	404 (0.91%)	2,450 (0.69%)	<0.001	404 (0.91%)	1,217 (0.69%)	<0.001
Hormone therapy	114 (0.26%)	853 (0.24%)	0.505	114 (0.26%)	433 (0.24%)	0.623
Statins	4,973 (11.22%)	32,379 (9.13%)	<0.001	4,973 (11.22%)	17,721 (9.99%)	<0.001
Antihypertensive	22,589 (50.94%)	154,670 (43.63%)	<0.001	22,589 (50.94%)	82,840 (46.71%)	<0.001
Sedative	5,781 (13.04%)	30,251 (8.53%)	<0.001	5,781 (13.04%)	15,843 (8.93%)	<0.001
Bisphosphonates	127 (0.29%)	562 (0.16%)	<0.001	127 (0.29%)	364 (0.21%)	0.001

COPD: chronic obstructive pulmonary disease, PVD: peripheral vascular disease

NSAID: Non-Steroidal Anti-Inflammatory Drug
