# Supplementary material for: Proton pump inhibitor use and risk of hip fracture in patients with type 2 diabetes
Source: Sci Rep. 2020 Aug 21;10:14081. doi: 10.1038/s41598-020-70712-9 (PMC7443131; doi:10.1038/s41598-020-70712-9)
Supplement: Supplementary file 2 — Supplementary file2 [file 41598_2020_70712_MOESM2_ESM.pdf]

Supplementary Table 2. Comorbidity-stratified analysis and medication use of hip fracture risk

	N	Total person-year	Fracture No.	Incident rate	Crude HR (95% CI)	p-value	Adjust HR (95% CI)	p-value
**Comorbidity (Ref = no)**								
Hypertension	94,937	442,590	1,376 (1.45%)	3.11	1.68 (1.55–1.82)	<0.001	0.85 (0.77–0.95)	0.004
Stroke	15,477	65,371	469 (3.03%)	7.17	3.50 (3.17–3.87)	<0.001	1.75 (1.56–1.96)	<0.001
Asthma	7,517	33,256	157 (2.09%)	4.72	2.05 (1.75–2.41)	<0.001	1.04 (0.85–1.27)	0.712
COPD	19,575	85,710	407 (2.08%)	4.75	2.20 (1.97–2.44)	<0.001	1.13 (0.98–1.29)	0.096
Myocardial Infarction	3,602	15,805	67 (1.86%)	4.24	1.81 (1.42–2.30)	<0.001	1.05 (0.82–1.35)	0.710
Chronic Heart Failure	4,226	16,648	118 (2.79%)	7.09	3.10 (2.58–3.73)	<0.001	1.23 (1.02–1.50)	0.034
Dementia	138	551	3 (2.17%)	5.45	2.32 (0.75–7.19)	0.144	0.64 (0.21–1.99)	0.442
Depression	992	4,485	26 (2.62%)	5.80	2.45 (1.67–3.61)	<0.001	1.46 (0.99–2.16)	0.058
Schizophrenia	1,717	8,069	20 (1.16%)	2.48	1.04 (0.67–1.62)	0.859	1.55 (0.98–2.45)	0.060
Chronic renal failure	2,074	7,987	70 (3.38%)	8.76	3.80 (3.00–4.82)	<0.001	1.93 (1.50–2.46)	<0.001
PVD	1,351	6,154	27 (2.00%)	4.39	1.85 (1.27–2.71)	0.001	1.01 (0.69–1.48)	0.964
Rheumatoid arthritis	1,086	5,021	28 (2.58%)	5.58	2.36 (1.63–3.42)	<0.001	1.50 (1.03–2.19)	0.034
**Medication (Ref = non-user)**								
NSAID	111,841	526,050	1,390 (1.24%)	2.64	1.25 (1.15–1.35)	<0.001	1.00 (0.92–1.08)	0.953
Corticosteroids	65,307	302,068	869 (1.33%)	2.88	1.32 (1.21–1.43)	<0.001	0.98 (0.90–1.07)	0.696
Anticoagulants	19,751	86,703	461 (2.33%)	5.32	2.52 (2.28–2.79)	<0.001	1.26 (1.13–1.40)	<0.001
Diuretics	70,556	321,316	1,136 (1.61%)	3.54	1.89 (1.75–2.05)	<0.001	1.10 (1.00–1.20)	0.043
Antipsychotic	15,638	70,039	263 (1.68%)	3.76	1.65 (1.45–1.87)	<0.001	1.10 (0.96–1.26)	0.179
Thyroxine	1,621	7,591	16 (0.99%)	2.11	0.88 (0.54–1.44)	0.620	0.73 (0.44–1.19)	0.203
Hormone therapy	547	2,419	6 (1.10%)	2.48	1.05 (0.47–2.33)	0.915	0.66 (0.30–1.47)	0.310
Statins	22,694	108,535	211 (0.93%)	1.94	0.80 (0.69–0.92)	0.002	0.61 (0.52–0.70)	<0.001
Antihypertensive	105,429	491,095	1,522 (1.44%)	3.10	1.77 (1.64–1.92)	<0.001	1.00 (0.89–1.11)	0.955
Sedative	21,624	96,943	430 (1.99%)	4.44	2.05 (1.84–2.27)	<0.001	1.33 (1.19–1.48)	<0.001
Bisphosphonates	491	2,015	16 (3.26%)	7.94	3.38 (2.07–5.52)	<0.001	1.35 (0.82–2.21)	0.236

95%

CI:

95%

confidence interval, HR: relative hazard ratio, Ref.: reference

COPD: chronic obstructive pulmonary disease, PVD: peripheral vascular disease

NSAID: Non-Steroidal Anti-Inflammatory Drug
